# Penetrating injury of the hand with a door handle: a case report

**DOI:** 10.1186/1752-1947-2-377

**Published:** 2008-12-08

**Authors:** Kiran Singisetti, Michail Kokkinakis, Nanjappa Shankar

**Affiliations:** 1 Department of Trauma and Orthopaedics, Queen Elizabeth Hospital, Gateshead, NE9 6SX, UK

## Abstract

**Introduction:**

Penetrating injuries of the hand with various sharp objects have previously been reported. In this report we describe an unusual penetrating injury of the hand caused by a door handle.

**Case presentation:**

A 32-year-old woman presented with a door handle stuck into her hand. After a preliminary assessment she was immediately taken to theatre. Broad spectrum antibiotics were administered along with tetanus toxoid. Soft tissue, including neurovascular integrity, was assessed and confirmed during the operation. She had a good functional recovery at follow-up.

**Conclusion:**

A door handle can occasionally cause a penetrating injury of the hand which should be treated with early intervention, including a careful assessment of soft tissue and neurovascular integrity.

## Introduction

Doors and door related injuries have been reported in the literature but most often they are related to crushing of the fingers, particularly the tips. Minor injuries often involve bruising or a blackened fingernail when a hand is caught in the latch end of the door as someone carelessly closes it. Sometimes the injury is more serious such as degloving injuries or fractures [1]. Injuries related to door handles have been very infrequently reported. Pointed door handles have been reported to pose a significant risk of ocular and peri-ocular injuries among young children [2,3]. We report an unusual injury where a door handle was found completely embedded into the hand of a woman.

## Case presentation

**Figure 1:**
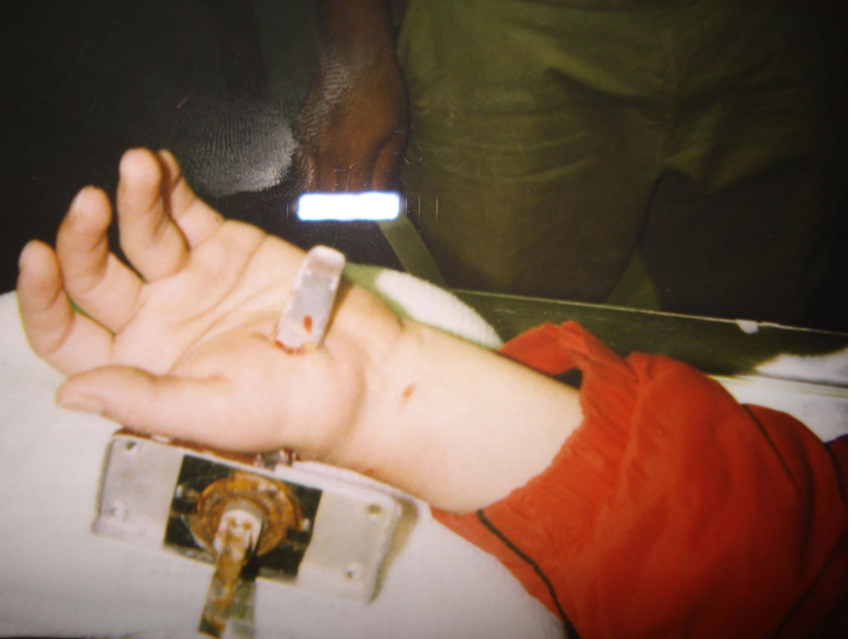
Clinical photograph of hand.

**Figure 2:**
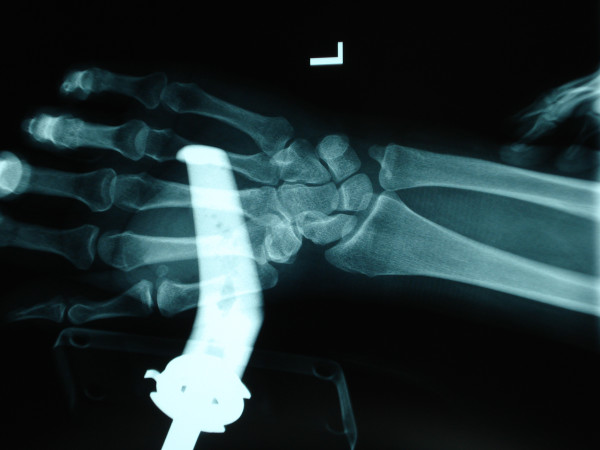
Antero-posterior radiograph of hand.

**Figure 3:**
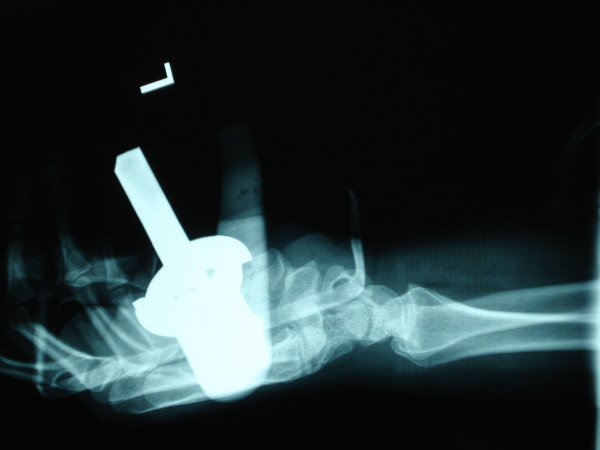
Lateral radiograph of hand.

A 32-year-old woman presented with a door handle stuck into her left non-dominant hand. While trying to move an old door at home, it accidentally fell on her injuring the left hand. The handle had passed through the dorsal aspect of the first web space and had exited though the volar aspect of the hand. She was able to move her fingers and had no distal neurovascular deficit at presentation. The capillary refill time was less then 2 seconds and the fingers showed no discoloration. There was minimal bleeding from the wound and there were no other associated injuries. After a preliminary assessment she was immediately taken to theatre. Clinical photographs (Figure 1) and radiographs (Figure 2 and 3) were taken preoperatively. Tetanus toxoid and intravenous antibiotics were administered. A tourniquet was used at appropriate pressure to aid the procedure. Intraoperative findings showed some 'paint' debris in the deeper tissues and the wound was washed with normal saline. The wound involved the capsule of the first metacarpophalangeal joint, which was repaired with 4-0 vicryl sutures. The integrity of tendons, nerves and blood vessels was confirmed and appropriate debridement and washout was performed. The wound was then reviewed after 48 hours and a delayed primary closure performed. The patient had regained good hand function and the wound healed unremarkably at follow-up.

## Discussion

Penetrating injuries of the hand are generally encountered in cases of gunshot and stab injuries. To our knowledge, a penetrating injury by a door handle has not previously been described in the literature. A debridement procedure and antibiotic cover are generally the essence of primary management. The use of broad spectrum antibiotics such as third generation cephalosporins is preferred but this may need to be modified depending on individual situations [4]. If surgical removal of a foreign body or surgical exploration of a puncture wound is decided upon, it must be performed under adequate anaesthesia and tourniquet control [5]. Attention to the integrity of the neurovascular and musculotendinous structures is important to improve the final outcome. Early mobilisation and physiotherapy helps in the return of functional activities after such injuries [6].

## Conclusion

Objects such as door handles can occasionally cause penetrating injuries of the hand. Such injuries should be managed with meticulous early surgical exploration and care to check neurovascular integrity.

## Consent

Written informed consent was obtained from the patient for publication of this case report and accompanying images. A copy of the written consent is available for review by the Editor-in-Chief of this journal.

## Competing interests

The authors declare that they have no competing interests.

## Authors' contributions

KS was involved with the initial writing of the manuscript including the literature search. MK was involved with the initial management of the patient and reviewed the manuscript. NS was the senior author responsible for the management of the patient.
